# *catena*-Poly[[[[*N*′-(4-cyano­benzyl­idene)nicotinohydrazide]silver(I)]-μ-[*N*′-4-cyano­benzyl­idene)nicotinohydrazide]] hexa­fluoridoarsenate]

**DOI:** 10.1107/S1600536809026907

**Published:** 2009-07-15

**Authors:** Cao-Yuan Niu, Ai-Min Ning, Chun-Hong Kou, Yong He, Zhi-Qiang Fen

**Affiliations:** a College of Sciences, Henan Agricultural University, Zhengzhou 450002, People’s Republic of China

## Abstract

In the title compound, {[Ag(C_14_H_10_N_4_O)_2_]AsF_6_}_*n*_, the Ag^I^ ion is coordinated by two N atoms from two different pyridyl rings and one N atom from one carbonitrile group of three different *N*′-(4-cyano­benzyl­idene)nicotinohydrazide ligands in a distorted T-shaped geometry. The Ag—N_carbonitrile_ bond distance is significant longer than those of Ag—N_pyrid­yl_. The bond angles around the Ag^I^ atom are also not in line with those in an ideal T-shaped geometry. One type of ligand acts as the bridge that connects Ag^I^ atoms into chains along [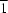
01]. These chains are linked to each other *via* N—H⋯O hydrogen bonds and Ag⋯O inter­actions with an Ag⋯O separation of 2.869 (2) Å. In addition, the [AsF_6_]^−^ counter-anions are linked to the hydrazone groups through N—H⋯F hydrogen bonds. Four of the F atoms of the [AsF_6_]^−^ anion are disordered over two sets of sites with occupancies of 0.732 (9) and 0.268 (9).

## Related literature

For background to silver coordination polymers, see: Dong *et al.* (2004 ▶); Niu *et al.* (2007 ▶, 2008 ▶); Sumby & Hardie (2005 ▶); Vatsadze *et al.* (2004 ▶); Zheng *et al.* (2003 ▶).
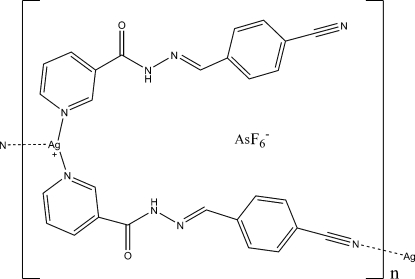


## Experimental

### 

#### Crystal data

[Ag(C_14_H_10_N_4_O)_2_]AsF_6_
*M* *_r_* = 797.31
Monoclinic, 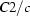

*a* = 22.3785 (15) Å
*b* = 13.7662 (9) Å
*c* = 19.8482 (14) Å
β = 99.948 (1)°
*V* = 6022.6 (7) Å^3^
*Z* = 8
Mo *K*α radiation
μ = 1.84 mm^−1^
*T* = 173 K
0.52 × 0.12 × 0.11 mm

#### Data collection

Bruker APEXII CCD area-detector diffractometer
Absorption correction: multi-scan (*SADABS*; Sheldrick, 1996 ▶) *T*_min_ = 0.448, *T*_max_ = 0.823
19207 measured reflections
6896 independent reflections
5017 reflections with *I* > 2σ(*I*)
*R*_int_ = 0.028

#### Refinement

*R*[*F* ^2^ > 2σ(*F* ^2^)] = 0.038
*wR*(*F* ^2^) = 0.105
*S* = 1.02
6896 reflections
460 parameters
96 restraints
H atoms treated by a mixture of independent and constrained refinement
Δρ_max_ = 0.63 e Å^−3^
Δρ_min_ = −0.47 e Å^−3^

### 

Data collection: *SMART* (Siemens, 1996 ▶); cell refinement: *SAINT* (Siemens, 1996 ▶); data reduction: *SAINT*; program(s) used to solve structure: *SHELXS97* (Sheldrick, 2008 ▶); program(s) used to refine structure: *SHELXL97* (Sheldrick, 2008 ▶); molecular graphics: *SHELXTL* (Sheldrick, 2008 ▶) and *DIAMOND* (Brandenburg, 2005 ▶); software used to prepare material for publication: *SHELXL97*.

## Supplementary Material

Crystal structure: contains datablocks I, global. DOI: 10.1107/S1600536809026907/jh2084sup1.cif

Structure factors: contains datablocks I. DOI: 10.1107/S1600536809026907/jh2084Isup2.hkl

Additional supplementary materials:  crystallographic information; 3D view; checkCIF report

## Figures and Tables

**Table:** 

Ag1—N5	2.183 (3)
Ag1—N1	2.204 (2)
Ag1—N4^i^	2.458 (3)

**Table:** 

N5—Ag1—N1	156.68 (9)
N5—Ag1—N4^i^	108.09 (11)
N1—Ag1—N4^i^	92.87 (11)

Symmetry code: (i) 
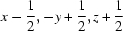
.

**Table 2:** Hydrogen-bond geometry (Å, °)

*D*—H⋯*A*	*D*—H	H⋯*A*	*D*⋯*A*	*D*—H⋯*A*
N6—H29⋯O1^ii^	0.868 (19)	2.13 (2)	2.976 (4)	165 (4)
N2—H28⋯F5	0.868 (19)	2.19 (2)	3.003 (4)	157 (3)

Symmetry code: (ii) 
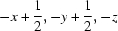
.

**Fig. 1.**
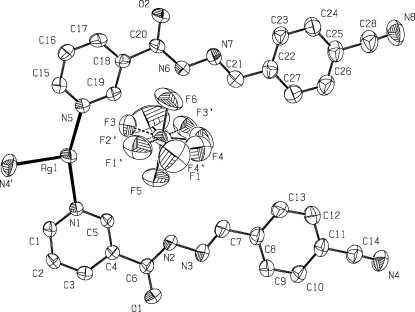
A view of the AgI coordination environment in the polymeric structure of (I), showing the atom-labelling scheme. Displacement ellipsoids are drawn at the 50% probability level and H atoms are omitted for clarity. [Symmetry codes: (i) x - 1/2, -y + 1/2, z + 1/2.]

**Fig. 2.**
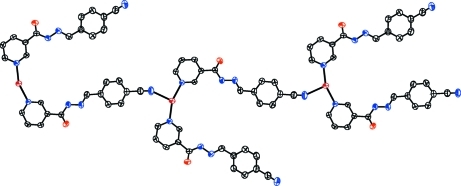
A e llipsoid diagram at the 50% probability level showing the one-dimensional chain. All counteranions and H atoms have been omitted for clarity.

**Fig. 3.**
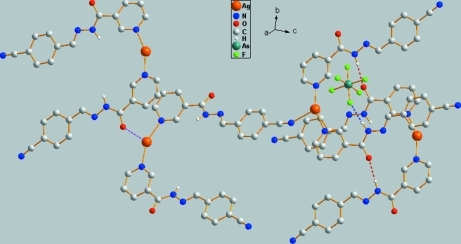
A diagram showing the intermolecular hydrogen bondings indicated by blue and red dashed lines and Ag···O interactions indicated by pink dashed lines.

## supplementary crystallographic information

### Comment

In the title compound, (I), the central silver ion is coordinated by two nitrogen atoms from two pyridyl rings of two different ligands (N1, N5) and one nitrogen atom from one carbonitrile group of another ligand [N4^i^. Symmetry codes: (i) *x* - 1/2, -*y* + 1/2, *z* + 1/2] forming a slightly distorted T-shaped coordination enviroment (Fig. 1). The bond angle of N1—Ag1—N5 is shorter than 180 ° and bond angles of N1—Ag1—N4^i^ and N5—Ag1—N4^i^ are larger than the right angle (Table 1). The N—Ag bond distances for pyridyl rings are 2.183 (3) and 2.204 (2) Å, which are smaller than N—Ag bond distance for carbonitrile group.

The compound 4-Cyanobenzylidene nicotinohydrazide act as the µ_2_-bridging ligands only by coordinations of pyridyl and carbonitrile nitrogen atoms. Each of these bridging ligands connects two silver atoms together by one pyridyl nitrogen atom N1 and one carbonitrile nitrogen atom N8 to form a one-dimensional chain along [-1,0,1] direction. The shortest distance between two silver atoms in one chain is about 16.28 Å. Meanwhile, the rest half of all ligands acting as terminal ligands are coordinated to silver atoms in chains only through pyridyl nitrogen atoms with the carbonitrile nitrogen atoms uncoordinating (Fig.2).

There are hydrogen bondings between uncoordinating groups, including pyridyl rings of terminal ligands and all hydrazone groups and counteranions. On one hand, counteranions AsF_6_^-^ are attached to ligands of chains by N—H···F hydrogen bondings (Table 2). On the other hand, there are also N—H···O hydrogen bondings (Table 2) between two neighbouring antiparallel chains. In addition to these intermolecular hydrogen bondings, there are weak Ag···O interactions between one oxygen atom O1 of the terminal ligand in one chain and one silver atom in the neighbouring chain with the Ag···O separations of 2.876 (2) Å (Fig. 3).

### Experimental

A solution of AgAsF_6_ (0.032 g, 0.1 mmol) in CH_3_OH (10 ml) was carefully layered on a CH_3_OH/CHCl_3_ solution (5 ml/10 ml) of 4-Cyanobenzylidene nicotinohydrazide (0.025 g, 0.1 mmol) in a straight glass tube. About ten days later, colourless single crystals suitable for X-ray analysis were obtained (yield about 30%). One very strong bonds at 699 cm^-1^ in the IR spectra was assigned to AsF_6_^-^.

### Refinement

C-bound H atoms were placed in calculated positions and refined using a riding model [C—H = 0.95 Å and *U*_iso_(H) = 1.2*U*_eq_(C)]. The N-bound H atoms were first introduced in calculated positions, and then thier positions and displacement parameters were refined with the N—H bond distance to 0.88 (2) Å. Four F atoms (F1—F4) of the hexafluoroarsenate anions are disordered over two positions, with maximum and minimum occupancies of 0.732 (9) and 0.268 (9), respectively. All As—F bond lengths were restrained to 1.70 (2) Å. Restraints of displacement parameters for six F or disordered F atoms were also performed. The final difference Fourier map had a max and min electron density of 0.63 and -0.47 e Å^-3^, respectively, but were otherwise featureless.

### Crystal data

**Table:**

[Ag(C_14_H_10_N_4_O)_2_]AsF_6_	*F*(000) = 3152
*M**_r_* = 797.31	*D*_x_ = 1.759 Mg m^−^^3^
Monoclinic, *C*2/*c*	Mo *K*α radiation, λ = 0.71073 Å
Hall symbol: -C 2yc	Cell parameters from 5377 reflections
*a* = 22.3785 (15) Å	θ = 2.1–27.5°
*b* = 13.7662 (9) Å	µ = 1.84 mm^−^^1^
*c* = 19.8482 (14) Å	*T* = 173 K
β = 99.948 (1)°	Prism, colourless
*V* = 6022.6 (7) Å^3^	0.52 × 0.12 × 0.11 mm
*Z* = 8	

### Data collection

**Table:**

Bruker APEXII CCD area-detector diffractometer	6896 independent reflections
Radiation source: fine-focus sealed tube	5017 reflections with *I* > 2σ(*I*)
graphite	*R*_int_ = 0.028
φ and ω scans	θ_max_ = 27.5°, θ_min_ = 2.1°
Absorption correction: multi-scan (*SADABS*; Sheldrick, 1996)	*h* = −29→18
*T*_min_ = 0.448, *T*_max_ = 0.823	*k* = −17→17
19207 measured reflections	*l* = −25→25

### Refinement

**Table:**

Refinement on *F*^2^	Primary atom site location: structure-invariant direct methods
Least-squares matrix: full	Secondary atom site location: difference Fourier map
*R*[*F*^2^ > 2σ(*F*^2^)] = 0.038	Hydrogen site location: inferred from neighbouring sites
*wR*(*F*^2^) = 0.105	H atoms treated by a mixture of independent and constrained refinement
*S* = 1.02	*w* = 1/[σ^2^(*F*_o_^2^) + (0.0526*P*)^2^ + 5.7237*P*] where *P* = (*F*_o_^2^ + 2*F*_c_^2^)/3
6896 reflections	(Δ/σ)_max_ = 0.001
460 parameters	Δρ_max_ = 0.63 e Å^−^^3^
96 restraints	Δρ_min_ = −0.46 e Å^−^^3^

### Special details

**Table:**

Geometry. All e.s.d.'s (except the e.s.d. in the dihedral angle between two l.s. planes) are estimated using the full covariance matrix. The cell e.s.d.'s are taken into account individually in the estimation of e.s.d.'s in distances, angles and torsion angles; correlations between e.s.d.'s in cell parameters are only used when they are defined by crystal symmetry. An approximate (isotropic) treatment of cell e.s.d.'s is used for estimating e.s.d.'s involving l.s. planes.
Refinement. Refinement of *F*^2^ against ALL reflections. The weighted *R*-factor *wR* and goodness of fit *S* are based on *F*^2^, conventional *R*-factors *R* are based on *F*, with *F* set to zero for negative *F*^2^. The threshold expression of *F*^2^ > σ(*F*^2^) is used only for calculating *R*-factors(gt) *etc*. and is not relevant to the choice of reflections for refinement. *R*-factors based on *F*^2^ are statistically about twice as large as those based on *F*, and *R*- factors based on ALL data will be even larger.

### Fractional atomic coordinates and isotropic or equivalent isotropic displacement parameters (Å^2^)

**Table:**

	*x*	*y*	*z*	*U*_iso_*/*U*_eq_	Occ. (<1)
Ag1	0.183850 (14)	0.187891 (18)	0.197856 (15)	0.05547 (11)	
N1	0.20655 (13)	0.32823 (17)	0.15519 (13)	0.0404 (6)	
N2	0.33194 (14)	0.3474 (2)	0.02005 (14)	0.0471 (7)	
N3	0.37007 (13)	0.3480 (2)	−0.02752 (14)	0.0480 (7)	
N4	0.61376 (15)	0.2368 (3)	−0.23488 (16)	0.0667 (9)	
N5	0.17227 (13)	0.03059 (19)	0.20139 (13)	0.0435 (6)	
N6	0.26690 (13)	−0.16253 (19)	0.09119 (14)	0.0440 (6)	
N7	0.30676 (13)	−0.2188 (2)	0.06251 (14)	0.0452 (6)	
N8	0.5343 (2)	−0.4267 (3)	−0.1374 (2)	0.0990 (15)	
O1	0.29409 (11)	0.49652 (17)	−0.01123 (12)	0.0540 (6)	
O2	0.25137 (12)	−0.28036 (16)	0.16611 (13)	0.0569 (6)	
C1	0.19143 (15)	0.4121 (2)	0.18267 (16)	0.0426 (7)	
H1	0.1669	0.4098	0.2172	0.051*	
C2	0.21021 (17)	0.5008 (2)	0.16271 (17)	0.0478 (8)	
H2	0.1992	0.5585	0.1838	0.057*	
C3	0.24503 (16)	0.5056 (2)	0.11201 (16)	0.0448 (8)	
H3	0.2582	0.5665	0.0975	0.054*	
C4	0.26041 (14)	0.4200 (2)	0.08263 (15)	0.0365 (7)	
C5	0.24032 (15)	0.3332 (2)	0.10548 (16)	0.0389 (7)	
H5	0.2508	0.2745	0.0852	0.047*	
C6	0.29680 (15)	0.4257 (2)	0.02645 (15)	0.0400 (7)	
C7	0.40093 (16)	0.2709 (3)	−0.02952 (17)	0.0513 (8)	
H7	0.3955	0.2179	−0.0005	0.062*	
C8	0.44496 (15)	0.2627 (3)	−0.07618 (16)	0.0461 (8)	
C9	0.46234 (17)	0.3429 (3)	−0.11026 (18)	0.0499 (8)	
H9	0.4440	0.4042	−0.1055	0.060*	
C10	0.50556 (18)	0.3345 (3)	−0.15064 (18)	0.0528 (9)	
H10	0.5178	0.3901	−0.1732	0.063*	
C11	0.53175 (15)	0.2446 (3)	−0.15883 (16)	0.0476 (8)	
C12	0.51410 (18)	0.1635 (3)	−0.1266 (2)	0.0594 (10)	
H12	0.5312	0.1018	−0.1330	0.071*	
C13	0.47112 (18)	0.1734 (3)	−0.0847 (2)	0.0588 (10)	
H13	0.4594	0.1181	−0.0615	0.071*	
C14	0.57804 (17)	0.2385 (3)	−0.20097 (18)	0.0527 (9)	
C15	0.13563 (17)	−0.0091 (2)	0.24079 (17)	0.0491 (8)	
H15	0.1117	0.0327	0.2633	0.059*	
C16	0.13129 (18)	−0.1077 (3)	0.24988 (19)	0.0538 (9)	
H16	0.1051	−0.1333	0.2784	0.065*	
C17	0.16547 (17)	−0.1682 (2)	0.21708 (18)	0.0473 (8)	
H17	0.1633	−0.2366	0.2228	0.057*	
C18	0.20314 (14)	−0.1297 (2)	0.17564 (15)	0.0370 (7)	
C19	0.20477 (15)	−0.0293 (2)	0.16906 (15)	0.0397 (7)	
H19	0.2301	−0.0021	0.1402	0.048*	
C20	0.24264 (15)	−0.1984 (2)	0.14389 (16)	0.0398 (7)	
C21	0.33262 (17)	−0.1765 (2)	0.01809 (18)	0.0488 (8)	
H21	0.3241	−0.1102	0.0072	0.059*	
C22	0.37552 (16)	−0.2300 (3)	−0.01634 (17)	0.0473 (8)	
C23	0.38639 (17)	−0.3282 (3)	−0.00388 (19)	0.0507 (8)	
H23	0.3654	−0.3614	0.0269	0.061*	
C24	0.42693 (18)	−0.3782 (3)	−0.03525 (19)	0.0552 (9)	
H24	0.4341	−0.4453	−0.0260	0.066*	
C25	0.45761 (17)	−0.3300 (3)	−0.0808 (2)	0.0556 (9)	
C26	0.44726 (19)	−0.2317 (3)	−0.0942 (2)	0.0625 (10)	
H26	0.4682	−0.1987	−0.1251	0.075*	
C27	0.40629 (19)	−0.1822 (3)	−0.0622 (2)	0.0587 (10)	
H27	0.3990	−0.1152	−0.0715	0.070*	
C28	0.5002 (2)	−0.3825 (3)	−0.1133 (2)	0.0700 (12)	
As1	0.377276 (19)	0.09939 (3)	0.14799 (2)	0.05697 (13)	
F5	0.38907 (15)	0.21973 (18)	0.13618 (16)	0.1020 (9)	
F6	0.36110 (16)	−0.01902 (19)	0.1542 (2)	0.1292 (13)	
H28	0.3375 (17)	0.306 (2)	0.0537 (14)	0.056 (11)*	
H29	0.2555 (17)	−0.1086 (19)	0.0702 (18)	0.061 (12)*	
F1	0.3247 (3)	0.1084 (5)	0.0751 (3)	0.142 (3)	0.732 (9)
F2	0.3208 (3)	0.1242 (3)	0.1925 (3)	0.111 (2)	0.732 (9)
F3	0.4252 (4)	0.0990 (7)	0.2174 (5)	0.203 (4)	0.732 (9)
F4	0.4308 (3)	0.0776 (4)	0.1007 (5)	0.135 (3)	0.732 (9)
F1'	0.3034 (4)	0.1259 (9)	0.1417 (10)	0.111 (5)	0.268 (9)
F2'	0.3871 (8)	0.1196 (8)	0.2320 (4)	0.095 (5)	0.268 (9)
F3'	0.4516 (4)	0.0732 (7)	0.1563 (8)	0.084 (4)	0.268 (9)
F4'	0.3710 (9)	0.0775 (9)	0.0641 (5)	0.116 (5)	0.268 (9)

### Atomic displacement parameters (Å^2^)

**Table:**

	*U*^11^	*U*^22^	*U*^33^	*U*^12^	*U*^13^	*U*^23^
Ag1	0.0725 (2)	0.03016 (14)	0.0723 (2)	0.00011 (12)	0.03658 (16)	0.00469 (11)
N1	0.0519 (16)	0.0292 (13)	0.0455 (15)	−0.0014 (11)	0.0234 (13)	0.0001 (10)
N2	0.0587 (18)	0.0446 (15)	0.0450 (16)	0.0072 (14)	0.0290 (14)	0.0084 (13)
N3	0.0510 (17)	0.0533 (17)	0.0455 (15)	0.0038 (14)	0.0248 (13)	0.0021 (13)
N4	0.063 (2)	0.081 (2)	0.066 (2)	0.0065 (18)	0.0362 (18)	0.0071 (18)
N5	0.0556 (17)	0.0335 (14)	0.0455 (15)	0.0012 (12)	0.0209 (13)	0.0030 (11)
N6	0.0540 (17)	0.0341 (14)	0.0469 (16)	0.0066 (12)	0.0175 (14)	0.0016 (12)
N7	0.0505 (17)	0.0408 (14)	0.0466 (15)	0.0024 (13)	0.0150 (13)	−0.0058 (12)
N8	0.104 (3)	0.080 (3)	0.134 (4)	−0.010 (2)	0.081 (3)	−0.022 (3)
O1	0.0690 (17)	0.0442 (13)	0.0556 (14)	0.0016 (12)	0.0298 (13)	0.0130 (11)
O2	0.0758 (18)	0.0302 (11)	0.0705 (16)	0.0041 (11)	0.0285 (14)	0.0048 (11)
C1	0.0487 (19)	0.0373 (17)	0.0474 (18)	0.0036 (14)	0.0241 (15)	0.0002 (13)
C2	0.066 (2)	0.0305 (15)	0.0521 (19)	0.0043 (15)	0.0249 (17)	−0.0060 (14)
C3	0.058 (2)	0.0293 (15)	0.0505 (19)	−0.0016 (14)	0.0196 (16)	0.0020 (13)
C4	0.0427 (17)	0.0330 (15)	0.0356 (15)	−0.0004 (13)	0.0119 (13)	0.0004 (12)
C5	0.0505 (19)	0.0277 (14)	0.0429 (17)	−0.0005 (13)	0.0202 (15)	−0.0033 (12)
C6	0.0471 (19)	0.0370 (16)	0.0393 (16)	−0.0033 (14)	0.0169 (14)	−0.0004 (13)
C7	0.054 (2)	0.055 (2)	0.050 (2)	0.0019 (17)	0.0259 (17)	0.0060 (16)
C8	0.0439 (19)	0.056 (2)	0.0419 (17)	0.0036 (16)	0.0161 (15)	0.0004 (15)
C9	0.057 (2)	0.0452 (18)	0.053 (2)	0.0041 (16)	0.0240 (17)	−0.0017 (15)
C10	0.062 (2)	0.050 (2)	0.052 (2)	−0.0039 (17)	0.0267 (18)	0.0020 (16)
C11	0.0433 (19)	0.059 (2)	0.0446 (18)	0.0019 (16)	0.0179 (15)	−0.0010 (15)
C12	0.060 (2)	0.055 (2)	0.070 (2)	0.0133 (18)	0.031 (2)	0.0047 (18)
C13	0.062 (2)	0.053 (2)	0.070 (2)	0.0099 (18)	0.034 (2)	0.0157 (18)
C14	0.052 (2)	0.060 (2)	0.050 (2)	0.0052 (17)	0.0173 (17)	0.0032 (17)
C15	0.059 (2)	0.0436 (18)	0.0493 (19)	0.0054 (16)	0.0234 (17)	0.0022 (15)
C16	0.060 (2)	0.048 (2)	0.062 (2)	−0.0014 (17)	0.0319 (19)	0.0104 (16)
C17	0.056 (2)	0.0322 (16)	0.057 (2)	−0.0058 (14)	0.0179 (17)	0.0073 (14)
C18	0.0437 (18)	0.0300 (14)	0.0378 (16)	−0.0026 (13)	0.0086 (14)	0.0019 (12)
C19	0.0498 (19)	0.0324 (15)	0.0399 (16)	−0.0027 (13)	0.0164 (14)	0.0021 (12)
C20	0.0467 (19)	0.0278 (15)	0.0457 (17)	−0.0014 (13)	0.0099 (15)	−0.0021 (12)
C21	0.055 (2)	0.0429 (18)	0.0496 (19)	0.0016 (16)	0.0121 (17)	−0.0012 (15)
C22	0.0473 (19)	0.0509 (19)	0.0449 (18)	−0.0051 (16)	0.0113 (15)	−0.0066 (15)
C23	0.051 (2)	0.052 (2)	0.052 (2)	−0.0068 (16)	0.0190 (17)	−0.0040 (16)
C24	0.057 (2)	0.051 (2)	0.062 (2)	−0.0013 (17)	0.0216 (18)	−0.0078 (17)
C25	0.048 (2)	0.063 (2)	0.060 (2)	−0.0091 (18)	0.0211 (18)	−0.0159 (18)
C26	0.067 (3)	0.067 (3)	0.060 (2)	−0.013 (2)	0.029 (2)	−0.0030 (19)
C27	0.065 (3)	0.051 (2)	0.064 (2)	−0.0047 (18)	0.022 (2)	−0.0003 (17)
C28	0.066 (3)	0.068 (3)	0.084 (3)	−0.014 (2)	0.037 (2)	−0.014 (2)
As1	0.0619 (3)	0.03366 (19)	0.0799 (3)	−0.00267 (16)	0.0248 (2)	0.00097 (16)
F5	0.140 (3)	0.0476 (14)	0.123 (2)	−0.0164 (15)	0.034 (2)	0.0104 (14)
F6	0.139 (3)	0.0433 (14)	0.224 (4)	−0.0122 (16)	0.086 (3)	−0.0032 (19)
F1	0.126 (5)	0.177 (6)	0.109 (4)	−0.018 (4)	−0.025 (3)	−0.016 (4)
F2	0.147 (5)	0.084 (3)	0.127 (4)	−0.013 (3)	0.092 (4)	−0.024 (3)
F3	0.152 (6)	0.254 (8)	0.177 (7)	−0.023 (6)	−0.046 (5)	0.065 (6)
F4	0.125 (5)	0.092 (3)	0.219 (7)	0.009 (3)	0.115 (5)	0.000 (4)
F1'	0.075 (6)	0.096 (7)	0.162 (11)	0.008 (6)	0.021 (7)	0.017 (8)
F2'	0.161 (10)	0.079 (6)	0.054 (5)	0.020 (6)	0.043 (6)	−0.004 (4)
F3'	0.055 (5)	0.066 (6)	0.132 (9)	0.006 (4)	0.021 (6)	0.000 (6)
F4'	0.161 (11)	0.093 (7)	0.085 (7)	−0.017 (7)	−0.003 (7)	−0.018 (6)

### Geometric parameters (Å, °)

**Table:**

Ag1—N5	2.183 (3)	C11—C12	1.378 (5)
Ag1—N1	2.204 (2)	C11—C14	1.442 (4)
Ag1—N4^i^	2.458 (3)	C12—C13	1.382 (5)
N1—C5	1.344 (4)	C12—H12	0.9500
N1—C1	1.345 (4)	C13—H13	0.9500
N2—C6	1.353 (4)	C15—C16	1.374 (5)
N2—N3	1.378 (3)	C15—H15	0.9500
N2—H28	0.868 (19)	C16—C17	1.370 (5)
N3—C7	1.271 (5)	C16—H16	0.9500
N4—C14	1.131 (4)	C17—C18	1.382 (4)
N4—Ag1^ii^	2.458 (3)	C17—H17	0.9500
N5—C19	1.335 (4)	C18—C19	1.389 (4)
N5—C15	1.343 (4)	C18—C20	1.506 (4)
N6—C20	1.353 (4)	C19—H19	0.9500
N6—N7	1.376 (4)	C21—C22	1.468 (5)
N6—H29	0.868 (19)	C21—H21	0.9500
N7—C21	1.275 (4)	C22—C23	1.389 (5)
N8—C28	1.144 (5)	C22—C27	1.397 (5)
O1—C6	1.224 (4)	C23—C24	1.371 (5)
O2—C20	1.214 (4)	C23—H23	0.9500
C1—C2	1.372 (4)	C24—C25	1.394 (5)
C1—H1	0.9500	C24—H24	0.9500
C2—C3	1.377 (4)	C25—C26	1.391 (6)
C2—H2	0.9500	C25—C28	1.436 (5)
C3—C4	1.384 (4)	C26—C27	1.381 (5)
C3—H3	0.9500	C26—H26	0.9500
C4—C5	1.381 (4)	C27—H27	0.9500
C4—C6	1.492 (4)	As1—F3	1.593 (6)
C5—H5	0.9500	As1—F2'	1.667 (8)
C7—C8	1.468 (4)	As1—F4	1.671 (4)
C7—H7	0.9500	As1—F4'	1.674 (9)
C8—C13	1.384 (5)	As1—F1'	1.675 (10)
C8—C9	1.386 (5)	As1—F6	1.679 (3)
C9—C10	1.363 (5)	As1—F3'	1.682 (8)
C9—H9	0.9500	As1—F2	1.696 (4)
C10—C11	1.392 (5)	As1—F5	1.700 (2)
C10—H10	0.9500	As1—F1	1.704 (5)
			
N5—Ag1—N1	156.68 (9)	N5—C19—C18	123.0 (3)
N5—Ag1—N4^i^	108.09 (11)	N5—C19—H19	118.5
N1—Ag1—N4^i^	92.87 (11)	C18—C19—H19	118.5
C5—N1—C1	117.8 (3)	O2—C20—N6	124.0 (3)
C5—N1—Ag1	121.53 (19)	O2—C20—C18	120.1 (3)
C1—N1—Ag1	120.4 (2)	N6—C20—C18	115.9 (3)
C6—N2—N3	119.9 (3)	N7—C21—C22	120.3 (3)
C6—N2—H28	117 (2)	N7—C21—H21	119.8
N3—N2—H28	120 (3)	C22—C21—H21	119.8
C7—N3—N2	114.9 (3)	C23—C22—C27	118.9 (3)
C14—N4—Ag1^ii^	153.9 (3)	C23—C22—C21	120.9 (3)
C19—N5—C15	117.8 (3)	C27—C22—C21	120.2 (3)
C19—N5—Ag1	121.3 (2)	C24—C23—C22	121.2 (3)
C15—N5—Ag1	120.7 (2)	C24—C23—H23	119.4
C20—N6—N7	119.3 (3)	C22—C23—H23	119.4
C20—N6—H29	124 (3)	C23—C24—C25	119.6 (4)
N7—N6—H29	116 (3)	C23—C24—H24	120.2
C21—N7—N6	115.7 (3)	C25—C24—H24	120.2
N1—C1—C2	122.4 (3)	C26—C25—C24	120.2 (3)
N1—C1—H1	118.8	C26—C25—C28	120.3 (4)
C2—C1—H1	118.8	C24—C25—C28	119.5 (4)
C1—C2—C3	119.6 (3)	C27—C26—C25	119.6 (4)
C1—C2—H2	120.2	C27—C26—H26	120.2
C3—C2—H2	120.2	C25—C26—H26	120.2
C2—C3—C4	118.7 (3)	C26—C27—C22	120.6 (4)
C2—C3—H3	120.7	C26—C27—H27	119.7
C4—C3—H3	120.7	C22—C27—H27	119.7
C5—C4—C3	118.7 (3)	N8—C28—C25	177.5 (5)
C5—C4—C6	122.8 (3)	F3—As1—F4	92.5 (4)
C3—C4—C6	118.5 (3)	F2'—As1—F4	127.4 (6)
N1—C5—C4	122.8 (3)	F3—As1—F4'	142.0 (7)
N1—C5—H5	118.6	F2'—As1—F4'	177.2 (7)
C4—C5—H5	118.6	F4—As1—F4'	49.8 (6)
O1—C6—N2	123.1 (3)	F3—As1—F1'	125.0 (7)
O1—C6—C4	121.5 (3)	F2'—As1—F1'	89.8 (6)
N2—C6—C4	115.4 (3)	F4—As1—F1'	142.2 (6)
N3—C7—C8	120.7 (3)	F4'—As1—F1'	93.0 (7)
N3—C7—H7	119.6	F3—As1—F6	93.1 (3)
C8—C7—H7	119.6	F2'—As1—F6	94.7 (4)
C13—C8—C9	119.2 (3)	F4—As1—F6	92.9 (2)
C13—C8—C7	119.3 (3)	F4'—As1—F6	85.2 (4)
C9—C8—C7	121.5 (3)	F1'—As1—F6	89.7 (4)
C10—C9—C8	120.5 (3)	F3—As1—F3'	53.7 (5)
C10—C9—H9	119.8	F2'—As1—F3'	88.9 (6)
C8—C9—H9	119.8	F4'—As1—F3'	88.3 (6)
C9—C10—C11	120.0 (3)	F1'—As1—F3'	178.7 (7)
C9—C10—H10	120.0	F6—As1—F3'	90.4 (4)
C11—C10—H10	120.0	F3—As1—F2	90.1 (4)
C12—C11—C10	120.4 (3)	F2'—As1—F2	55.1 (5)
C12—C11—C14	121.0 (3)	F4—As1—F2	177.1 (3)
C10—C11—C14	118.6 (3)	F4'—As1—F2	127.7 (6)
C11—C12—C13	119.0 (3)	F6—As1—F2	88.16 (19)
C11—C12—H12	120.5	F3'—As1—F2	143.6 (5)
C13—C12—H12	120.5	F3—As1—F5	91.5 (3)
C12—C13—C8	120.9 (3)	F2'—As1—F5	88.8 (4)
C12—C13—H13	119.5	F4—As1—F5	87.4 (2)
C8—C13—H13	119.5	F4'—As1—F5	91.5 (4)
N4—C14—C11	177.6 (4)	F1'—As1—F5	87.3 (4)
N5—C15—C16	122.9 (3)	F6—As1—F5	175.38 (19)
N5—C15—H15	118.6	F3'—As1—F5	92.8 (4)
C16—C15—H15	118.6	F2—As1—F5	91.32 (19)
C17—C16—C15	118.7 (3)	F3—As1—F1	175.8 (4)
C17—C16—H16	120.6	F2'—As1—F1	142.2 (5)
C15—C16—H16	120.6	F4—As1—F1	89.6 (3)
C16—C17—C18	119.8 (3)	F1'—As1—F1	52.7 (6)
C16—C17—H17	120.1	F6—As1—F1	90.4 (3)
C18—C17—H17	120.1	F3'—As1—F1	128.6 (5)
C17—C18—C19	117.8 (3)	F2—As1—F1	87.7 (3)
C17—C18—C20	118.0 (3)	F5—As1—F1	85.0 (2)
C19—C18—C20	124.0 (3)		
			
N5—Ag1—N1—C5	−17.9 (4)	C10—C11—C12—C13	−1.6 (6)
N4^i^—Ag1—N1—C5	−172.2 (3)	C14—C11—C12—C13	177.7 (4)
N5—Ag1—N1—C1	168.2 (3)	C11—C12—C13—C8	1.4 (6)
N4^i^—Ag1—N1—C1	13.8 (3)	C9—C8—C13—C12	0.0 (6)
C6—N2—N3—C7	−179.8 (3)	C7—C8—C13—C12	−178.0 (4)
N1—Ag1—N5—C19	27.0 (4)	C19—N5—C15—C16	1.2 (5)
N4^i^—Ag1—N5—C19	180.0 (2)	Ag1—N5—C15—C16	−173.6 (3)
N1—Ag1—N5—C15	−158.4 (3)	N5—C15—C16—C17	−0.4 (6)
N4^i^—Ag1—N5—C15	−5.4 (3)	C15—C16—C17—C18	−0.3 (6)
C20—N6—N7—C21	−172.9 (3)	C16—C17—C18—C19	0.1 (5)
C5—N1—C1—C2	−1.3 (5)	C16—C17—C18—C20	176.5 (3)
Ag1—N1—C1—C2	172.9 (3)	C15—N5—C19—C18	−1.4 (5)
N1—C1—C2—C3	1.1 (6)	Ag1—N5—C19—C18	173.4 (2)
C1—C2—C3—C4	−0.3 (5)	C17—C18—C19—N5	0.7 (5)
C2—C3—C4—C5	−0.1 (5)	C20—C18—C19—N5	−175.4 (3)
C2—C3—C4—C6	178.4 (3)	N7—N6—C20—O2	−3.8 (5)
C1—N1—C5—C4	0.9 (5)	N7—N6—C20—C18	175.7 (3)
Ag1—N1—C5—C4	−173.2 (2)	C17—C18—C20—O2	−16.5 (5)
C3—C4—C5—N1	−0.2 (5)	C19—C18—C20—O2	159.6 (3)
C6—C4—C5—N1	−178.7 (3)	C17—C18—C20—N6	164.0 (3)
N3—N2—C6—O1	4.6 (5)	C19—C18—C20—N6	−19.9 (5)
N3—N2—C6—C4	−176.0 (3)	N6—N7—C21—C22	−179.3 (3)
C5—C4—C6—O1	150.3 (3)	N7—C21—C22—C23	3.3 (5)
C3—C4—C6—O1	−28.2 (5)	N7—C21—C22—C27	−176.5 (3)
C5—C4—C6—N2	−29.0 (5)	C27—C22—C23—C24	0.6 (6)
C3—C4—C6—N2	152.4 (3)	C21—C22—C23—C24	−179.2 (3)
N2—N3—C7—C8	−177.9 (3)	C22—C23—C24—C25	−0.4 (6)
N3—C7—C8—C13	−170.4 (4)	C23—C24—C25—C26	0.2 (6)
N3—C7—C8—C9	11.7 (6)	C23—C24—C25—C28	179.8 (4)
C13—C8—C9—C10	−1.3 (6)	C24—C25—C26—C27	−0.2 (6)
C7—C8—C9—C10	176.7 (4)	C28—C25—C26—C27	−179.7 (4)
C8—C9—C10—C11	1.2 (6)	C25—C26—C27—C22	0.4 (6)
C9—C10—C11—C12	0.3 (6)	C23—C22—C27—C26	−0.6 (6)
C9—C10—C11—C14	−179.0 (4)	C21—C22—C27—C26	179.2 (4)

Symmetry codes: (i) *x*−1/2, −*y*+1/2, *z*+1/2; (ii) *x*+1/2, −*y*+1/2, *z*−1/2.

### Hydrogen-bond geometry (Å, °)

**Table:**

*D*—H···*A*	*D*—H	H···*A*	*D*···*A*	*D*—H···*A*
N6—H29···O1^iii^	0.87 (2)	2.13 (2)	2.976 (4)	165 (4)
N2—H28···F5	0.87 (2)	2.19 (2)	3.003 (4)	157 (3)

Symmetry codes: (iii) −*x*+1/2, −*y*+1/2, −*z*.
